# Whole Genome Resequencing of 20 Accessions of Rice Landraces Reveals Javanica Genomic Structure Variation and Allelic Genotypes of a Grain Weight Gene TGW2

**DOI:** 10.3389/fpls.2022.857435

**Published:** 2022-04-25

**Authors:** Weixiong Long, Lihua Luo, Laiyang Luo, Weibiao Xu, Yonghui Li, Yaohui Cai, Hongwei Xie

**Affiliations:** Jiangxi Super-Rice Research and Development Center, Jiangxi Academy of Agricultural Sciences, National Engineering Center for Rice, Nanchang, China

**Keywords:** *Oryza sativa* javanica, SNPs/InDels, variant calling, specific variation, grain shapes

## Abstract

The landraces preserved by indigenous worldwide exhibited larger variation in the phenotypes and adaption to different environments, which suggests that they comprise rich resources and can be served as a gene pool for rice improvement. Despite extensive studies on cultivated rice, the variations and relationships between landraces and modern cultivated rice remain unclear. In this study, a total of 20 varieties that include 10 *Oryza* javanica collected from different countries worldwide and 10 *Oryza* indica from China were genotyped and yielded a sum of 99.9-Gb resequencing raw data. With the genomic sequence of the japonica cultivar Nipponbare as a reference, the following genetic features of single-nucleotide polymorphism (SNP) ranged from 861,177 to 1,044,617, insertion–deletion polymorphisms (InDels) ranged from 164,018 to 211,135, and structural variation (SV) ranged from 3,313 to 4,959 were identified in *Oryza* javanica. Variation between the two subspecies was also determined that 584,104 SNPs, 75,351 InDels, 104,606 SNPs, and 19,872 InDels specific to *Oryza* indica and *Oryza* javanica, respectively. Furthermore, Gene Ontology (GO) and KEGG of *Oryza* javanica-specific SNP-related genes revealed that they participated in DNA metabolic process, DNA replication, and DNA integration. The sequence variation and candidate grain shape-related gene *TGW2* were identified through Fst and sweep selective analysis. Hap4 of *TGW2* is performed better than others. The whole genome sequence data and genetic variation information illustrated in this study will serve as an important gene pool for molecular breeding and facilitate genetic analysis of *Oryza* javanica varieties.

## Introduction

The knowledge of the pattern of the variation in the rice species is a prerequisite for rice improvement as it helps breeders in choosing suitable breeding strategies for their breeding goals. Rice landraces were the lineages evolved *via* selective breeding by farmers in a time of long-term domestication (Pusadee et al., 2009). Wild relatives and landraces exhibited wide adaptation to various environments, which provides valuable and useful genetic resources for rice improvement (Sang and Ge, 2013; Dwivedi et al., 2016). Compared with modern cultivars, traditional rice landraces preserved by indigenous were under urgent need of protection and systematic evaluation to unveil new genes or QTLs for higher yield, higher resistance, and more friendly to environments (Ram et al., 2007; Pusadee et al., 2009; Hour et al., 2020). Hence, the investigation of the whole genome variation and allelic variation for grain shapes of these landraces was the paramount step for rice breeding programs.

*Oryza sativa* ssp. javanica is a large grain landrace that exhibited long and wide grains compared to *O. sativa* ssp. indica and *O. sativa* ssp. japonica (Eizenga et al., 2019). Some researchers proposed the hypothesis that *O. sativa* ssp. japonica derived from *O. sativa* ssp. javanica because of the larger standardized alleles and higher mutation rates showed by *O.* japonica (Garris et al., 2005). However, the previous studies for rice grain shapes concentrated on Indica (Fan et al., 2006; Weng et al., 2008; Li et al., 2011; Wang et al., 2012; Liu et al., 2017; Zhao et al., 2018), and the reports about genetic control of big grain in javanica have not been described, let alone the genetic differentiation of the subspecies. Earlier studies for *O. sativa* ssp. javanica mainly focused on the application of heterosis (Peng et al., 2008).

With the rapid development of sequencing technology, the high-quality rice reference genome and population resequencing provided an unparalleled convenience to explore the genome-wide variation exhibited among the landraces (Chen et al., 2021). The application of rice genomics could cost-effectively provide dense SNP markers and InDel markers (Davey et al., 2011). High-throughput genotyping based on SNP/InDel markers could provide exhaustive genetic information due to their abundance and uniform distribution throughout the genome. These markers could help with population structure analysis, genetic map construction, and gene mapped cloning (Long et al., 2020a). SNP markers had been widely used to characterize the rice population structure and identify the candidate gene/QTL for favor traits (Long et al., 2020b). InDel variation dispersed throughout the rice genome could develop InDel markers for identifying the varieties or species and function as SSR markers (Hu et al., 2020; Hechanova et al., 2021). Furthermore, genomic analysis of diverse genotypes such as wild relatives, landraces, cultivars, and modern rice was expected to provide new light on domestications, selection sweeps of specific genomic regions, and evolutions of grain shapes (Choi et al., 2020; Lin et al., 2020). Nowadays, long and wide grain javanica rice had evolved from their short, narrow grains progenitors such as *Oryza rufipogon* over thousand years of cultivation, domestication, and natural selection (Huang et al., 2012). The investigation of the complex grain shapes contributed by many genes remained interesting and challenging.

In this study, we aimed at understanding the genetic basis of big grains of *O. sativa* ssp. javanica by whole genome deep resequencing and comparative genomic analysis using 20 rice varieties that include 10 *O. sativa* ssp. indica and 10 *O. sativa* ssp. javanica with the reference genome of Nipponbare genome. We identified the whole genome SNP, InDel, and structure variation among the 20 rice accessions, and we also identified the common SNP and InDel variation between *O. sativa* ssp. indica and *O. sativa* ssp. javanica. Furthermore, private SNPs and InDels of javanica were identified, and the Gene Ontology (GO) analysis of the private InDels associated genes was conducted. Then, we performed selection sweeps of the two subpopulation. Additionally, the allelic variation of genes that controlled rice grain shapes, which also represented the selection sweep, was performed. The information described here can provide novel observations for rice breeding and genetic analysis.

## Materials and Methods

### Plant Materials

**FIGURE 1:**
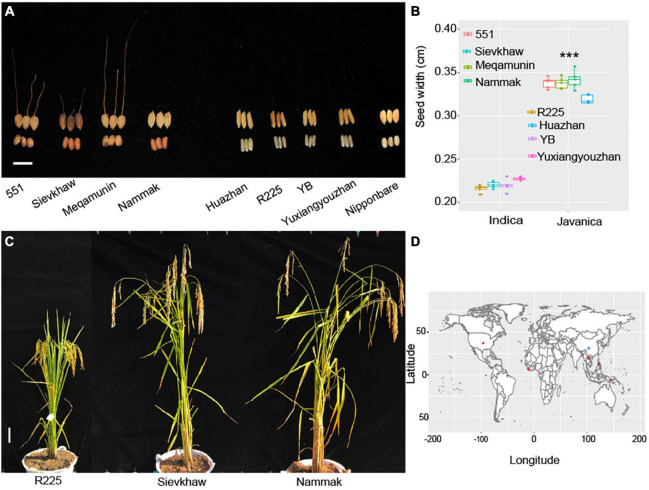
Phenotyping of the representative *Oryza* javanica and the source information. **(A)** Grain width of four *Oryza* javanica and four *Oryz*a indica, Nipponbare was used as control. **(B)** Boxplot of the grain width of two subspecies. ***p < 0.001. **(C)** The plant performance of Super indica rice R225 and two javanica varieties. **(D)** The world distribution of the 20 sequenced rice accession. Red color indicates the javanica accessions, blue color shows the indica rice varieties.

A total of 20 rice varieties, which include 10 *O. sativa* javanica and 10 *O. sativa* indica, were used for analysis in this study. The germplasm of *Oryza* javanica varieties was obtained from the International Rice Research Institute (IRRI, Philippines), and the *Oryza* indica core pool was collected from Jiangxi Super Rice Research and Development Center (JSRRDC). The geographical distribution of the rice varieties is collected in Figure 1. The plants’ performance of selected *Oryza* javanica and *Oryza* indica is documented in Figure 1. All of the *O. sativa* were planted in Nanchang, China. Each variety was planted for 5 rows and each row for 10 plants. Each variety was transplanted with a spacing of 30 cm. The randomized complete block design was carried out in this study.

### Whole Genome Resequencing and Mapping

For each rice accession, a single individual was used for whole genome resequencing. Genomic DNA was extracted from young leaves using a DNA Extraction Kit (Qiagen, Hilden, Germany), and sequencing libraries with an approximately 350-bp insert size were prepared. All samples were sequenced using the Illumina HiSeqTM 2500 by Biomarker Technologies (Beijing, China) according to the manufacturer’s instructions (Lv et al., 2020). In order to ensure the quality of information analysis, the raw reads were filtered based on the following criteria: (i) remove reads containing adapters, (ii) remove reads containing *N* > 5% (*N* represents base could not be determined), and (iii) remove reads where the *q* score (quality value) of over 50% bases of the read below 20. The clean reads were mapped to the reference genome of Nipponbare (Os-Nipponbare-Reference-IRGSP-1.0, MSU release 7) by using Burrows–Wheeler Alignment (BWA) software (v0.7.12) (Li and Durbin, 2009). The sequencing depth and coverage of each 100-kb window were calculated by SAMtools software (Li et al., 2009).

### Single-Nucleotide Polymorphism, Insertion–Deletion Polymorphism, and Structure Variation Detection

The alignment results were merged and converted into binary alignment map (BAM) files. Then, BAM out files were first sorted using Picard software, which was used to calculate the sequencing depth. For SNP and InDel variation identification, GATK (v3.8) software was used according to the following criteria (McKenna et al., 2010): the read depth is large than 10 and the quality score ≥50. Breakdancer was used to detect SVs that include insertions (Fan et al., 2014), larger deletions (>100 bp), inversion, intra-chromosomal rearrangements, and inter-chromosomal translocations based on mapped read pairs. The number of genomic variations in 100-kb sliding window size across the whole genome was calculated. The genomic distribution of SNPs, InDels, and SVs on each chromosome was visualized using Circos software (Krzywinski et al., 2009). The SnpEff tool was used to annotate the SNP and InDel and identify the large effect of variations (Cingolani et al., 2012).

### Shared and Private Variation Detection Between Indica and Javanica

To better identify the variation between the two subspecies, we intend to extract the shared SNPs/InDels variations to belong to *O. sativa* ssp. indica and *O. sativa* ssp. javanica, respectively. We divide the VCF file into two VCF files based on the subspecies samples. The private SNP/InDel variation is unique to one specific subspecies but not presented in the other subspecies were obtained using BCFtools (Danecek and McCarthy, 2017). To better understand the function of the private InDel-associated genes, we extracted the gene ID based on the location information from GFF file (Oryza_sativa.IRGSP-1.0.51.gff3) and performed gene ontology analysis. We also analyzed shared variation between *Oryza* indica and *Oryza* javanica.

### Population Structure and Linkage Disequilibrium Decay Analysis

GCTA software was used to conduct a PCA to estimate the number of subpopulations (Yang et al., 2011). Whole genome SNP was used to constructed neighbor-joining tree using SNPhylo software and visualized using the online web (Interactive Tree of Life, iTOL) (Lee et al., 2014). LD was calculated using PopLDdecay software (Zhang et al., 2019), the pairwise *r*^2^ was calculated for all the SNPs in a 50-kb window and averaged across the whole genome and 12 chromosomes separately.

### Fst, Pi, and Selective Sweep of the Subspecies

To evaluate the genetic relationship of the two subspecies, pairwise genetic differentiation (Fst) for SNPs along with all chromosomes between indica and javanica was calculated using VCFtools v0.1.10 and represented pictorially using Circos with 100-kb fixed window (Danecek et al., 2011). Nucleotide diversity (Π) is often applied to measure the degree of variability in a population. The selective sweeps determined for javanica and indica rice were identified using reduction of diversity (ROD) and fixation index (Fst), and windows with the top 5% of maximum Fst and maximum and minimum ROD values were considered as selection regions (Wang et al., 2018). To identify the grain shape selection-related genes, we subjected the selective sweep regions and located within the previously mapped rice grain shapes QTL regions, and the overlapped regions were considered as the grain shape selection regions (Miao et al., 2020). To better understand the grain shape genes between the subspecies, we conducted the haplotype analysis of the selective grain shape genes.

## Results

### Phenotyping of the Two Subspecies *Oryza* Indica and *Oryza* Javanica

The yield-related traits that include plant height, grain length, grain width, and panicle length of *Oryza* javanica show more than *Oryza* indica. The day to maturity of *Oryza* javanica exhibited longer than indica due to their photosensitivity. The average of four selected javanica rice’s width is 0.33 cm, which is almost 1.5-fold than that of *O. sativa* indica which selected as most cultivated in China (Figure 1).

### Whole Genome Resequencing and Reads Mapping

A total of 99.94-GB raw data that include 396,571,548 paired-end reads of 250 bp were generated from the 20 rice varieties with approximately 13 × depth (Table 1). The percentage of Q30 Phred quality score ranged from 86.48 to 89.05% with an average of 87.83% (Table 1). A total of 99.78-GB raw data were obtained after filtered, and the filtered reads were used for further analysis. The GC content of *Oryza* indica and *Oryza* javanica was 43.15 and 42.33%, respectively. The average of 88.11% of *Oryza* indica and 92.97% of *Oryza* tropic japonica clean reads were mapped to the Nipponbare genome, which covers 82.31 and 89.20% of the reference genome. The reads depth of each rice landraces based on 100-kb window was ranged from 0 to 180 and presented in Supplementary Figure 1. The 20 resequencing data generated in this study were submitted to the National Genomic Data Center with the BioProject number PRJNA763248.

**TABLE 1:** The resequencing information of 20 rice varieties.

Subspecies	Sequencing ID	Variety name	Raw reads	Clean reads	Ave_depth	Mapped (%)	SNP	InDel	SV
Indica	R01	R752	18,145,916	18,117,663	10	88.6	2,138,145	389,902	7,235
	R02	Hefengzhan	16,732,881	16,707,308	9	88.67	2,186,224	394,344	6,790
	R03	R458	16,404,329	16,380,301	9	88.18	2,127,805	370,332	6,908
	R09	Yuxiangyouzhan	19,594,960	19,564,428	11	88.89	2,259,101	412,756	7,530
	R10	Haodali	20,437,203	20,406,924	11	88.68	2,195,047	404,504	7,642
	R11	XieqingzaoB	18,263,185	18,235,592	10	82.71	2,251,559	405,028	7,046
	R12	R225	17,742,018	17,714,419	10	83.54	2,237,723	405,144	7,469
	R16	Guinongzhan	18,355,001	18,326,915	9	84.45	2,135,555	373,548	6,895
	R17	YuetaiB	20,425,253	20,394,151	11	87.74	2,433,805	439,866	8,837
	R18	Huazhan	18,503,276	18,474,866	10	88.37	2,080,799	356,485	6,821
Javanica	R04	Qamuyan	17,117,976	17,093,051	10	93.15	863,573	164,018	3,475
	R05	13B	18,255,101	18,224,806	11	92.39	911,759	174,719	3,313
	R06	551	17,962,140	17,930,382	11	92.53	942,898	179,895	3,733
	R07	13494	23,082,133	23,282,528	14	92.23	950,923	182,814	4,089
	R08	Qipaprt 2	22,556,756	22,523,437	13	93.1	870,429	173,290	3,992
	R13	Nam mak	23,082,133	23,048,803	13	93.44	861,177	175,559	3,981
	R14	Siew khaw	22,976,506	22,939,888	13	92.58	1,044,617	211,135	4,959
	R15	11390	21,220,932	21,190,655	12	93.58	879,135	173,524	3,928
	R19	Meqamunin 2	23,002,937	22,970,983	13	92.89	919,683	182,366	4,050
	R20	Bugel	22,474,942	22,444,383	14	93.78	861,530	170,263	3,908

### Single-Nucleotide Polymorphisms, Insertion–Deletion Polymorphisms, and Structural Variation Identification

**FIGURE 2:**
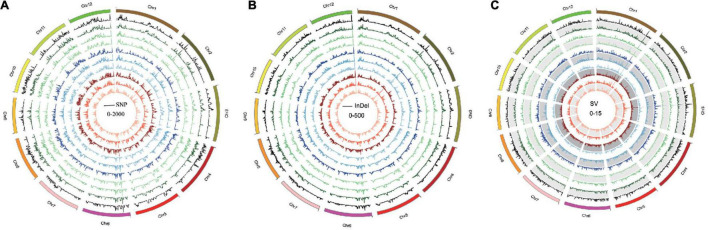
Genome-wide variation pattern of the 10 *Oryza* javanica. **(A)** The SNP variation distribution of 10 *Oryza* javanica among the genome. **(B)** The InDel variation of 10 *Oryza* javanica. **(C)**. The SV pattern of 10 *Oryza* javanica was characterized with Nipponbare reference genome.

Single-nucleotide polymorphisms, InDels, and SVs in each *Oryza* javanica variety were identified based on the reference genome (Table 1). Overall, all the *Oryza* indica showed a similar pattern of DNA variations, which were 2–3 times higher compared with the SNPs of *Oryza* javanica. The average numbers of SNPs for *Oryza* indica and *Oryza* javanica were 2,204,576 and 910,613, respectively. The InDels number was significantly lower than the SNP number in both subspecies. The InDels number of *Oryza* indica ranged from 356,485 to 439,866, with an average of 395,190. A relatively low InDels number that ranges from 164,059 to 211,176 was obtained in *Oryza* javanica. The number of SVs was quite fewer than that of SNPs and InDels in both species. The total number of SVs in *O. sativa* indica ranged from 6,790 to 8,837, while that ranged from 3,313 to 4,959 in *Oryza* javanica. The detailed SV type and distribution information of all the 20 rice accessions are listed in Supplementary Table 1. The densities of SNPs, InDels, and SVs in both subspecies showed a similar profile. The distribution of SNPs, InDels, and SVs on all 12 chromosomes of each subspecies in comparison with the reference genome is presented in Figure 2.

### Population Structure Analysis of the 20 Rice Accessions

**FIGURE 3:**
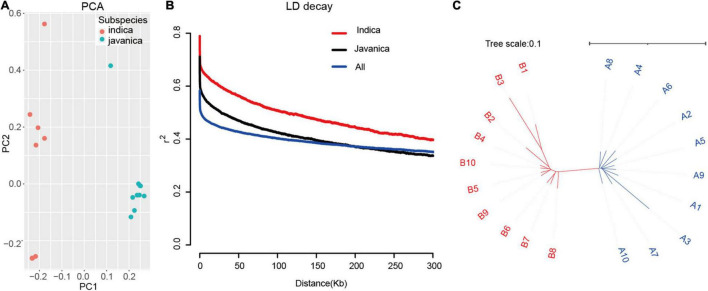
Population structure of the 20 rice varieties. **(A)** PCA of 20 rice accessions. Colors red and green indicate indica and javanica, respectively. **(B)** Decay of LD expressed as *r*^2^ as a function of inter-SNP distance for filtered MBML-intersect SNP, in the indica, javanica, and whole 20 population. **(C)** Unweighted neighbor-joining tree for genome-wide SNP, horizontal bar indicates distance by simple matching coefficient.

To understand the structure of the 20 rice accessions worldwide, PCA based on the whole genome SNP was conducted. The PCA results highly revealed that two main clusters correspond to two groups, indica and javanica. The phylogenetic tree was constructed using the maximum likelihood method clustered these varieties into two major groups, which are similar to the subpopulation identified by PCA (Figure 3). We examined LD decay in each subpopulation and all 20 rice accessions separately. As expected, the *r*^2^ value declined with the increasing physical distance between markers. LD extends to 300 kb for the indica group, which is a higher estimate than reported (McNally et al., 2009), while the LD is approximately 200 kb in javanica group. These results revealed that the utilization of these varieties had a slight advantage over that of other sets of japonica rice germplasm due to the few candidates in an LD block.

### Distribution of Shared and Private Single-Nucleotide Polymorphisms and Insertion–Deletion Polymorphisms of Each Subspecies

**FIGURE 4:**
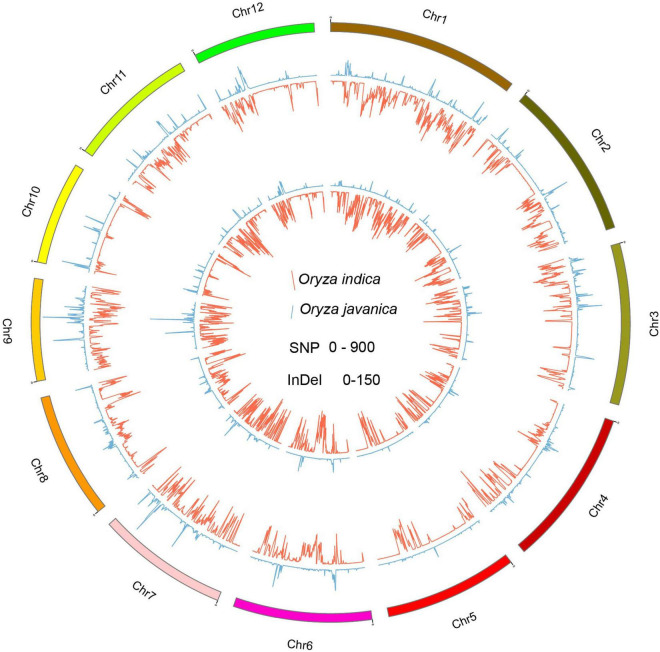
The genome-wide distribution of private variation between the two subspecies. The inside Circos indicates the private SNP variation pattern among the whole genome. The outside Circos shows the private InDel variation number distribution along the whole genome. Red indicates indica, and green means javanica.

To better understand the variation pattern of the subpopulation, the indica and javanica variations were conducted, respectively. A total number of 649,857 and 170,370 common SNPs were identified in *Oryza* indica and *Oryza* javanica varieties, respectively. Among them, a total number of 584,104 and 104,518 private SNP belong to *Oryza* indica and javanica, respectively. The largest number of common SNPs is derived from the two subspecies both located on chromosome 1, while the lowest number of common SNPs was detected on chromosomes 9 and 5 for *Oryza* indica and *Oryza* javanica, respectively (Supplementary Tables 2, 3). A total number of 28,751 and 23,726 common and private InDels were found in javanica varieties, respectively. Unlike the variation pattern of SNP in javanica, the largest number for common and private InDels was both located on chromosome 6 and the lowest number for common and private InDels was characterized on chromosomes 9 and 10, respectively. In addition, bioinformatic analysis using the resequencing data of 20 varieties revealed that the common InDels number in *Oryza* indica is 7–40 times for 100-kb windows higher than that in *Oryza* javanica. The common InDels number located on the whole chromosome ranged from 5,429 to 13,706, which was identified in *Oryza* indica (Supplementary Tables 2, 3). The extracted common SNPs and InDels density were plotted using Circos program (Figure 4). The Venn diagram for shared SNPs and InDels variation of two subspecies is presented in Supplementary Figure 2.^1^

### Gene Ontology Analysis of *Oryza* Javanica Varieties

**FIGURE 5:**
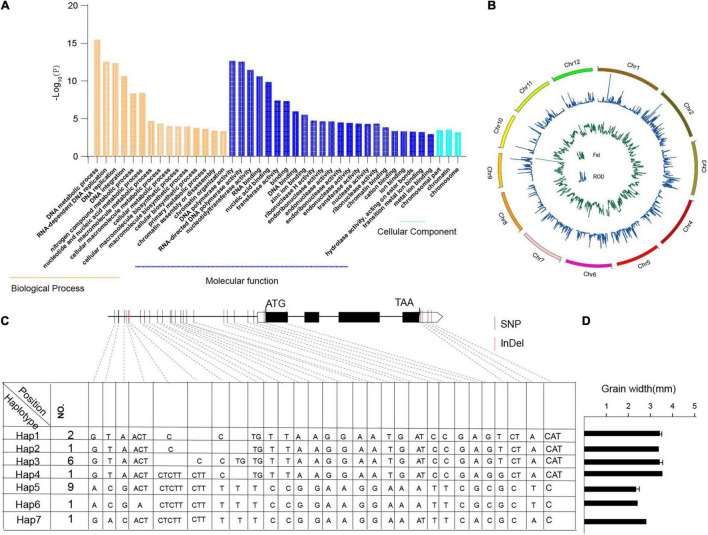
Gene Ontology (GO) analysis of javanica private genes and haplotype analysis of TGW2 in 20 rice accessions. **(A)** GO enrichment analysis of the javanica private variation-associated genes. **(B)**. Selective sweep in the two subspecies. The green line indicates the reduction of diversity of two subspecies; the blue line shows fixation index of the two subspecies. **(C)**. Sequence and allelic variation in *TGW2* among 20 rice accessions. Sequence and allelic variations in *TGW2* including promoters and coding sequence (CDS) from 10 *Oryza* javanica and 10 improved indica cultivars were analyzed. Hap1–Hap6 represents six different haplotypes of *TGW2*, and No. represents the number of rice accessions for each type. The position of start codon is considered as +1. In CDS region, the black box represents exons whereas the lines between boxes represent introns. Nucleotide polymorphisms are displayed at their corresponding positions. The black vertical line indicates SNPs while red vertical line stands for InDels. **(D)** The grain width of the sample which carried different haplotypes of TGW2. Hap7 was represented Nipponbare.

In this study, we identified 104,518 SNPs that were specific to *Oryza* javanica and not present in any *Oryza* indica, which affects about 4,852 genes (Supplementary Table 4). To further investigate their putative functions affected in javanica varieties compared to the subspecies indica, GO and KEGG enrichment analyses were performed (Figure 5A and Supplementary Table 4). Genes involved in biological processes such as “DNA metabolic process,” “RNA-dependent DNA replication,” “DNA replication,” and “DNA integration” were significantly enriched. Analysis at the molecular function level illustrated that “DNA polymerase activity,” “RNA-directed DNA polymerase activity,” and “nucleotidyltransferase activity” were overrepresented. These specific SNPs might be, to some extent, responsible for the contrasting grain shapes between *Oryza* indica and *Oryza* javanica.

### Divergence of the Javanica Rice Germplasm

The assessment of genetic diversity within the total accessions revealed a higher level of genetic diversity within the admix group compared to the indica and javanica subspecies. When the indica and japonica accessions were analyzed separately, the javanica varieties showed a lower level of polymorphism than that of *Oryza* indica (Supplementary Figure 3). However, the genetic diversity of *Oryza* javanica was higher than *Oryza* indica in some regions, such as chromosomes 1, 4, and 10. Moreover, the highest value of pi (0.0077 at chromosome 2) in *Oryza* javanica is also larger than that of *Oryza* indica (0.0073 on chromosome 11), which indicated that there was a greater variation in genetic diversity at some regions in the javanica group.

### Haplotype Analysis of the Grain Shape-Related Selective Sweep Genes

To find the selection evidence of the grain size genes, we scanned each sweep region generated by the linkage disequilibrium of the selection target and its surrounding loci, which is expected to affect the genetic diversity. In order to identify the grain shapes related to selective sweep genes, genome-wide Fst and ROD were conducted in the two subpopulations (Figure 5B). Following the criteria of top 5% sites of Fst and top 2% or bottle 2% site of ROD in the whole genome might be the candidate regions involved in selection (Supplementary Tables 5, 6). The overlapped candidate region was treated as grain shape-related genes. Finally, three candidate grain shape-related genes were detected, which shows significant differences between haplotypes (Supplementary Table 7 and Figure 5C). Haplotype analyses of the *TGW2* are presented in Figure 5D. Combining the phenotype of grain shape, the grain width score of Japonica is higher than that belong to Indica.

## Discussion

*Oryza sativa* independently domesticated rice that has been cultivated for more than 10,000 years, which is the predominant energy source for most people worldwide. The rice production should be doubled to meet the increased demand of population in 2050 according to FAO data (McClung, 2014). The successful application of heterosis in hybrid rice has dramatically improved rice productivity in the past years (Lin et al., 2020). However, indica rice is the predominant form of hybrid rice in China due to the high incompatibility of interspecies between indica and japonica (Huang et al., 2015). It is well known that heterosis of inter-subspecies is usually stronger than that of intra-subspecies, and the level of yield advantage ranked as indica/temperate japonica > indica/tropical japonica > temperate japonica/tropical japonica > indica/indica > japonica/japonica.

Genetic diversity is fundamentally important in the hybrid rice programs to breed heterotic rice hybrids because the accomplishment of heterosis is dependent on the genetic differences between parents (Zheng et al., 2020). Rice landraces are adapted to local environments and selected by the farmers for their better yield. Tropical japonica, also named javanica, were planted by indigenous in southeast and east Asia, Latin America, and Africa and represented by tall, large, and bold-grain bulu cultivars of Indonesia. However, these landraces are on the brink of extinction due to a lack of adequate attention. Characterization of these landraces for the desired trait can create new materials of big grain in rice.

Whole genome resequencing of the 10 *Oryza* javanica and 10 *Oryza* indica varieties and mapping with Nipponbare genome as a reference genome was conducted to discover genome-wide DNA variations. A total of 4,057,525 SNPs and 642,824 InDels were identified. When we analyzed the DNA polymorphism between indica and javanica, a total of 27,657 and 83,136 InDel are private to all javanica and indica, respectively, whereas a total of 539,268 and 853,249 SNPs are private to javanica and indica, respectively. A total number of 7,915 SNPs and 65,841 InDels shared by indica and javanica were detected.

Population structure of the 20 rice varieties results revealed that they distinctly belonging to two groups. The variation of *Oryza* javanica is shown a similar DNA sequence variation, which includes SNP, InDel, and SV. This study focused on genotyping by sequencing and genetic analysis of 10 rice landraces collected all over the world and 10 improved indica cultivar varieties that cultivated in China. GO was important bioinformatic tools which attempt to interpret the role of genes or proteins through the organization of controlled terms. Functional cataloging of the javanica private InDels-related genes using GO slim terms offered the facility to scrutiny the genome into categories describing cellular locations, biological process, and molecular functions. Among the private variation-associated genes, the most significantly detected GO term is rice DNA polymerase activity (GO:0034061), which plays important role in rice growth and development. DNA replication is a fundamental nuclear metabolic process.

Morphology and genetic background are quite different between modern improvement indica and landraces javanica through independent origins, long-term adaption to diverse environment, and selection for breeder’s preference. PCA results revealed that two subpopulations were obviously observed, which suggests the significant differentiation between the two subpopulations. Recently, Si et al. (2016) had identified a major quantitative trait locus, *GLW7*, encoding the plant-specific transcription factor OsSPL13, which is derived from *Oryza* javanica that can positively regulate cell size in the grain hull, which results in enhanced rice grain length and yield. Hence, the selective sweep gene related to grain shape between the two subpopulations drives our attention.

The overall present study deals with whole genome variations in javanica rice accessions by identifying total SNP and InDel polymorphisms, 10 javanica common SNP and InDel variation by comparing each javanica variety, and private SNP and InDel polymorphisms by comparing with indica rice accessions. The private InDel-associated genes participating in the cellular biosynthetic process and primary metabolic process were revealed by comparative analysis. The differentiation of indica and javanica rice associated with grain shape was identified in this study. This work had provided a groundbreaking work for utilizing the heterosis of inter-subspecies.

## Data Availability Statement

The datasets presented in this study can be found in online repositories. The names of the repository/repositories and accession number(s) can be found in the article/Supplementary Material.

## Author Contributions

WL, YL, and HX conceived the work and designed the experiments. LiL, LaL, and WX performed the phenotype collection. WL and HX analyzed the results. YC provided part of the fundings. All authors contributed to writing the manuscript and discussed the results.

## Conflict of Interest

The authors declare that the research was conducted in the absence of any commercial or financial relationships that could be construed as a potential conflict of interest.

## Publisher’s Note

All claims expressed in this article are solely those of the authors and do not necessarily represent those of their affiliated organizations, or those of the publisher, the editors and the reviewers. Any product that may be evaluated in this article, or claim that may be made by its manufacturer, is not guaranteed or endorsed by the publisher.

## Footnotes

1
http://bioinformatics.psb.ugent.be/webtools/Venn/

## References

[B1] Chen R. Deng Y. Ding Y. Guo J. Qiu J. Wang B. (2021). Rice functional genomics :decades’ efforts and roads ahead. *Sci. China Life Sci.* 65 33–92. 10.1007/s11427-02102024-0 34881420

[B2] Choi J. Y. Lye Z. N. Groen S. C. Dai X. Rughani P. Zaaijer S. (2020). Nanopore sequencing-based genome assembly and evolutionary genomics of circum-basmati rice. *Genome Biol.* 21:21. 10.1186/s13059-020-1938-2 32019604 PMC7001208

[B3] Cingolani P. Platts A. Wang le L. Coon M. Nguyen T. Wang L. (2012). A program for annotating and predicting the effects of single nucleotide polymorphisms, SnpEff: SNPs in the genome of *Drosophila* melanogaster strain w1118; iso-2; iso-3. *Fly (Austin)* 6 80–92. 10.4161/fly.19695 22728672 PMC3679285

[B4] Danecek P. McCarthy S. A. (2017). BCFtools/csq: haplotype-aware variant consequences. *Bioinformatics* 33 2037–2039. 10.1093/bioinformatics/btx100 28205675 PMC5870570

[B5] Danecek P. Auton A. Abecasis G. Albers C. A. Banks E. DePristo M. A. (2011). The variant call format and VCFtools. *Bioinformatics* 27 2156–2158. 10.1093/bioinformatics/btr330 21653522 PMC3137218

[B6] Davey J. W. Hohenlohe P. A. Etter P. D. Boone J. Q. Catchen J. M. Blaxter M. L. (2011). Genome-wide genetic marker discovery and genotyping using next-generation sequencing. *Nat. Rev. Genet.* 12 499–510. 10.1038/nrg3012 21681211

[B7] Dwivedi S. L. Ceccarelli S. Blair M. W. Upadhyaya H. D. Are A. K. Ortiz R. (2016). Landrace germplasm for improving yield and abiotic stress adaptation. *Trends Plant Sci.* 21 31–42. 10.1016/j.tplants.2015.10.012 26559599

[B8] Eizenga G. C. Jia M. H. Jackson A. K. Boykin D. L. Ali M. L. Shakiba E. (2019). Validation of yield component traits identified by genome-wide association mapping in a tropical japonica x tropical japonica rice biparental mapping population. *Plant Genome* 12 10.3835/plantgenome2018.04.0021 30951093 PMC12962355

[B9] Fan C. Xing Y. Mao H. Lu T. Han B. Xu C. (2006). GS3, a major QTL for grain length and weight and minor QTL for grain width and thickness in rice, encodes a putative transmembrane protein. *Theor. Appl. Genet.* 112 1164–1171. 10.1007/s00122-006-0218-1 16453132

[B10] Fan X. Abbott T. E. Larson D. Chen K. (2014). BreakDancer: identification of genomic structural variation from paired-end read mapping. *Curr. Protoc. Bioinformatics* 45 15.6.1–15.6.11. 10.1002/0471250953.bi1506s45 25152801 PMC4138716

[B11] Garris A. J. Tai T. H. Coburn J. Kresovich S. McCouch S. (2005). Genetic sructure and diversity in *Oryza sativa* L. *Genetics* 169 1631–1638. 15654106 10.1534/genetics.104.035642 PMC1449546

[B12] Hechanova S. L. Bhattarai K. Simon E. V. Clave G. Karunarathne P. Ahn E. K. (2021). Development of a genome-wide InDel marker set for allele discrimination between rice (*Oryza sativa*) and the other seven AA-genome *Oryza* species. *Sci. Rep.* 11:8962. 10.1038/s41598-021-88533-9 33903715 PMC8076200

[B13] Hour A. L. Hsieh W. H. Chang S. H. Wu Y. P. Chin H. S. Lin Y. R. (2020). Genetic diversity of landraces and improved varieties of rice (*Oryza sativa* L.) in taiwan. *Rice (N.Y.)* 13:82. 10.1186/s12284-020-00445-w 33315140 PMC7736384

[B14] Hu W. Zhou T. Wang P. Wang B. Song J. Han Z. (2020). Development of whole-genome agarose-resolvable lindel markers in rice. *Rice (N.Y.)* 13:1. 10.1186/s12284-019-0361-3 31907673 PMC6944724

[B15] Huang X. Kurata N. Wei X. Wang Z. X. Wang A. Zhao Q. (2012). A map of rice genome variation reveals the origin of cultivated rice. *Nature* 490 497–501. 10.1038/nature11532 23034647 PMC7518720

[B16] Huang X. Yang S. Gong J. Zhao Y. Feng Q. Gong H. (2015). Genomic analysis of hybrid rice varieties reveals numerous superior alleles that contribute to heterosis. *Nat. Commun.* 6:6258. 10.1038/ncomms7258 25651972 PMC4327311

[B17] Krzywinski M. Schein J. Birol I. Connors J. Gascoyne R. Horsman D. (2009). Circos: an information aesthetic for comparative genomics. *Genome Res.* 19 1639–1645. 10.1101/gr.092759.109 19541911 PMC2752132

[B18] Lee T. H. Guo H. Wang X. Kim C. Paterson A. H. (2014). SNPhylo: a pipeline to construct a phylogenetic tree from huge SNP data. *BMC Genomics* 15:162. 10.1186/1471-2164-15-162 24571581 PMC3945939

[B19] Li H. Durbin R. (2009). Fast and accurate short read alignment with Burrows-Wheeler transform. *Bioinformatics* 25 1754–1760. 10.1093/bioinformatics/btp324 19451168 PMC2705234

[B20] Li H. Handsaker B. Wysoker A. Fennell T. Ruan J. Homer N. (2009). The sequence alignment/map format and SAMtools. *Bioinformatics* 25 2078–2079. 10.1093/bioinformatics/btp352 19505943 PMC2723002

[B21] Li Y. Fan C. Xing Y. Jiang Y. Luo L. Sun L. (2011). Natural variation in GS5 plays an important role in regulating grain size and yield in rice. *Nat. Genet.* 43 1266–1269. 10.1038/ng.977 22019783

[B22] Lin Z. Qin P. Zhang X. Fu C. Deng H. Fu X. (2020). Divergent selection and genetic introgression shape the genome landscape of heterosis in hybrid rice. *Proc. Natl. Acad. Sci. U.S.A.* 117 4623–4631. 10.1073/pnas.1919086117 32071222 PMC7060695

[B23] Liu J. Chen J. Zheng X. Wu F. Lin Q. Heng Y. (2017). GW5 acts in the brassinosteroid signalling pathway to regulate grain width and weight in rice. *Nat. Plants* 3:17043. 10.1038/nplants.2017.43 28394310

[B24] Long W. Dan D. Yuan Z. Chen Y. Jin J. Yang W. (2020a). Deciphering the genetic basis of lodging resistance in wild rice *Oryza longistaminata*. *Front. Plant Sci.* 11:628. 10.3389/fpls.2020.00628 32547576 PMC7274161

[B25] Long W. Yuan Z. Fan F. Dan D. Pan G. Sun H. (2020b). Genome-wide association analysis of resistance to rice false smut. *Mol. Breed.* 40 10.1007/s11032-020-01130-y

[B26] Lv Q. Li W. Sun Z. Ouyang N. Jing X. He Q. (2020). Resequencing of 1,143 indica rice accessions reveals important genetic variations and different heterosis patterns. *Nat. Commun.* 11:4778. 10.1038/s41467-020-18608-0 32963241 PMC7508829

[B27] McClung C. R. (2014). Plant science. Making hunger yield. *Science* 344 699–700. 10.1126/science.1254135 24833378

[B28] McKenna A. Hanna M. Banks E. Sivachenko A. Cibulskis K. Kernytsky A. (2010). The genome analysis toolkit: a mapreduce framework for analyzing next-generation DNA sequencing data. *Genome Res.* 20 1297–1303. 10.1101/gr.107524.110 20644199 PMC2928508

[B29] McNally K. L. Childs K. L. Bohnert R. Davidson R. M. Zhao K. Ulat V. J. (2009). Genomewide SNP variation reveals relationships among landraces and modern varieties of rice. *Proc. Natl. Acad. Sci. U.S.A.* 30 12273–12278. 10.1073/pnas.0900992106 19597147 PMC2718348

[B30] Miao L. Yang S. Zhang K. He J. Wu C. Ren Y. (2020). Natural variation and selection in GmSWEET39 affect soybean seed oil content. *New Phytol.* 225 1651–1666. 10.1111/nph.16250 31596499 PMC7496907

[B31] Peng S. Khush G. S. Virk P. Tang Q. Zou Y. (2008). Progress in ideotype breeding to increase rice yield potential. *Field Crops Res.* 108 32–38. 10.1016/j.fcr.2008.04.001

[B32] Pusadee T. Jamjod S. Chiang Y. C. Rerkasem B. Schaal B. A. (2009). Genetic structure and isolation by distance in a landrace of Thai rice. *Proc. Natl. Acad. Sci. U.S.A.* 106 13880–13885. 10.1073/pnas.0906720106 19651617 PMC2728989

[B33] Ram S. G. Thiruvengadam V. Vinod K. K. (2007). Genetic diversity among cultivars, landraces and wild relatives of rice as revealed by microsatellite markers. *J. Appl. Genet.* 48 337–345. 10.1007/BF03195230 17998590

[B34] Sang T. Ge S. (2013). Understanding rice domestication and implications for cultivar improvement. *Curr. Opin. Plant. Biol.* 16 139–146. 10.1016/j.pbi.2013.03.003 23545218

[B35] Si L. Chen J. Huang X. Gong H. Luo J. Hou Q. (2016). OsSPL13 controls grain size in cultivated rice. *Nat. Genet.* 48 447–456. 10.1038/ng.3518 26950093

[B36] Wang S. Wu K. Yuan Q. Liu X. Liu Z. Lin X. (2012). Control of grain size, shape and quality by OsSPL16 in rice. *Nat. Genet.* 44 950–954. 10.1038/ng.2327 22729225

[B37] Wang W. Mauleon R. Hu Z. Chebotarov D. Tai S. Wu Z. (2018). Genomic variation in 3,010 diverse accessions of Asian cultivated rice. *Nature* 557 43–49. 10.1038/s41586-018-0063-9 29695866 PMC6784863

[B38] Weng J. Gu S. Wan X. Gao H. Guo T. Su N. (2008). Isolation and initial characterization of GW5, a major QTL associated with rice grain width and weight. *Cell Res.* 18 1199–1209. 10.1038/cr.2008.307 19015668

[B39] Yang J. Lee S. H. Goddard M. E. Visscher P. M. (2011). GCTA: a tool for genome-wide complex trait analysis. *Am. J. Hum. Genet.* 88 76–82. 10.1016/j.ajhg.2010.11.011 21167468 PMC3014363

[B40] Zhang C. Dong S. S. Xu J. Y. He W. M. Yang T. L. (2019). PopLDdecay: a fast and effective tool for linkage disequilibrium decay analysis based on variant call format files. *Bioinformatics* 35 1786–1788. 10.1093/bioinformatics/bty875 30321304

[B41] Zhao D. S. Li Q. F. Zhang C. Q. Zhang C. Yang Q. Q. Pan L. X. (2018). GS9 acts as a transcriptional activator to regulate rice grain shape and appearance quality. *Nat. Commun.* 9:1240. 10.1038/s41467-018-03616-y 29588443 PMC5869696

[B42] Zheng W. Ma Z. Zhao M. Xiao M. Zhao J. Wang C. (2020). Research and development strategies for hybrid japonica rice. *Rice (N.Y.)* 13:36. 10.1186/s12284-020-00398-0 32514748 PMC7280405

