# Supplementary figures and images for: Whole Genome Resequencing of 20 Accessions of Rice Landraces Reveals Javanica Genomic Structure Variation and Allelic Genotypes of a Grain Weight Gene TGW2
Source: Front Plant Sci. 2022 Apr 25;13:857435. doi: 10.3389/fpls.2022.857435 (PMC9083905; doi:10.3389/fpls.2022.857435)

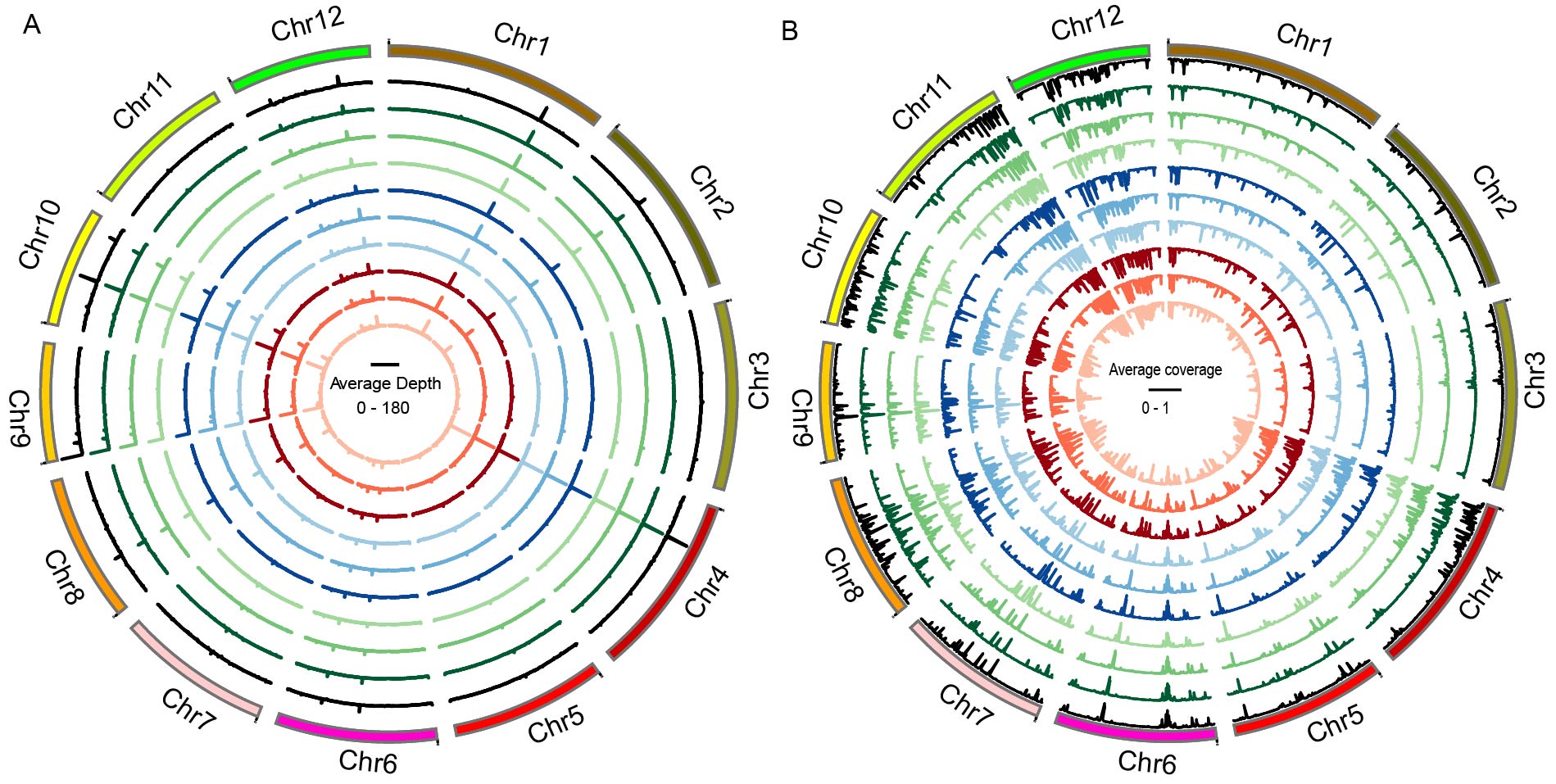

Supplement: Supplementary file 1 [file Image_1.JPEG]

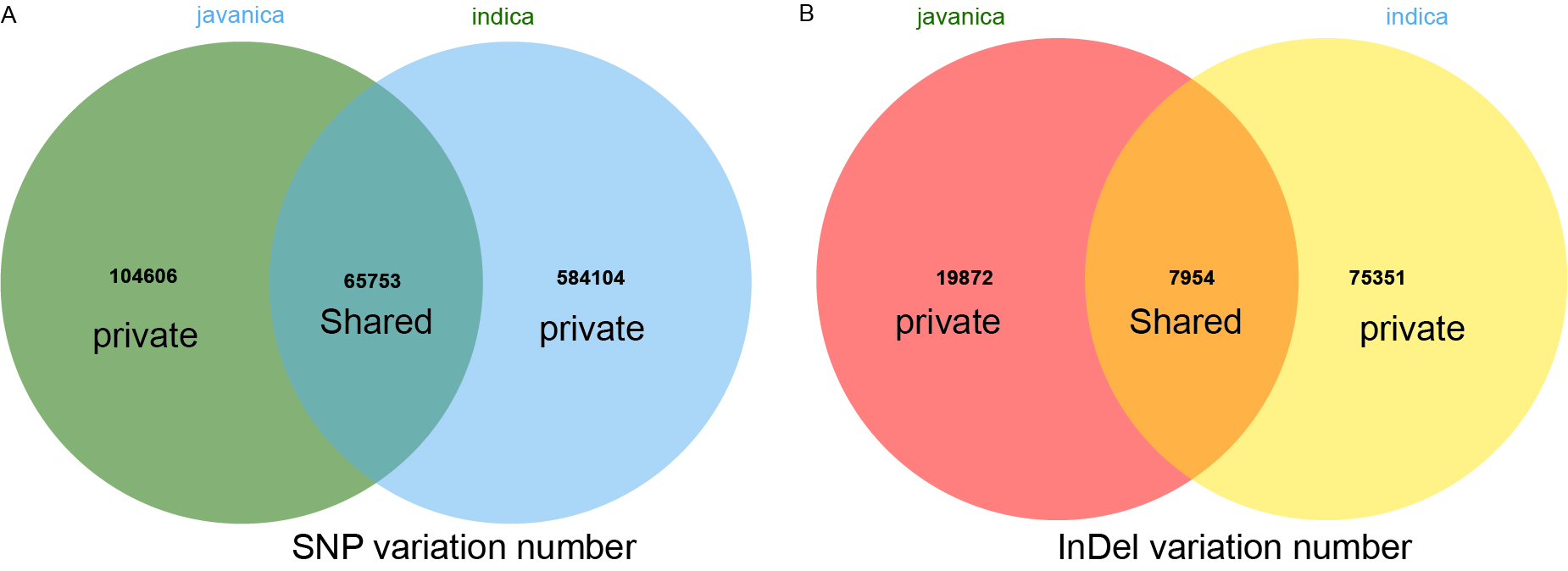

Supplement: Supplementary file 2 [file Image_2.JPEG]

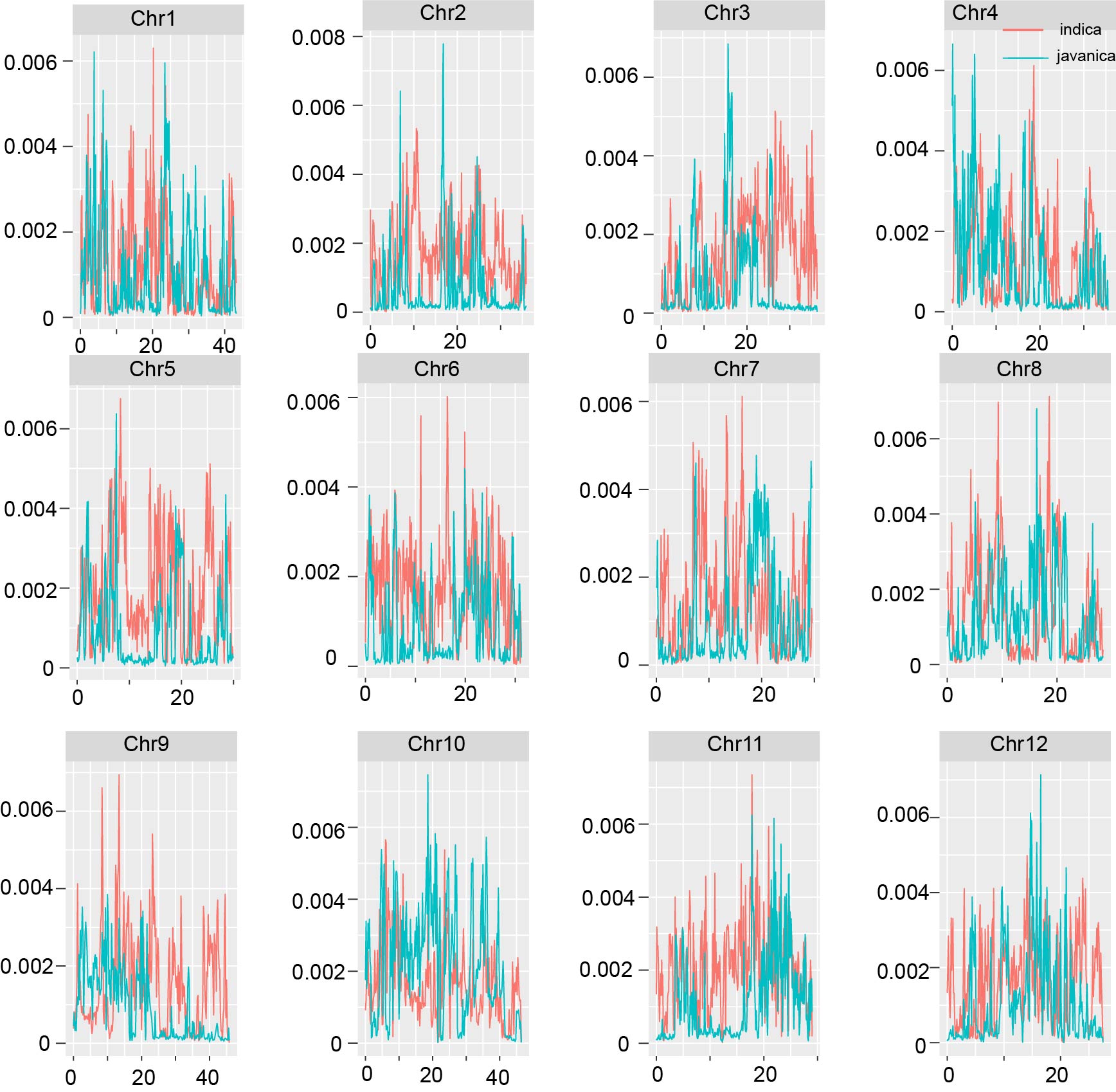

Supplement: Supplementary file 3 [file Image_3.JPEG]
